# Image analysis of anatomical traits in stalk transections of maize and other grasses

**DOI:** 10.1186/s13007-015-0070-x

**Published:** 2015-04-09

**Authors:** Sven Heckwolf, Marlies Heckwolf, Shawn M Kaeppler, Natalia de Leon, Edgar P Spalding

**Affiliations:** Department of Botany, University of Wisconsin-Madison, 430 Lincoln Drive, Madison, WI 53706 USA; Department of Agronomy, University of Wisconsin-Madison, 1575 Linden Drive, Madison, WI 53706 USA; DOE Great Lakes Bioenergy Research Center, 1552 University Avenue, Madison, WI 53706 USA

## Abstract

**Background:**

Grass stalks architecturally support leaves and reproductive structures, functionally support the transport of water and nutrients, and are harvested for multiple agricultural uses. Research on these basic and applied aspects of grass stalks would benefit from improved capabilities for measuring internal anatomical features. In particular, methods suitable for phenotyping populations of plants are needed.

**Results:**

To meet the need for large-scale measurements of stalk anatomy features, we developed custom image processing software that utilized a variety of global thresholding, local filtering, and feature detection methods to measure rind thickness, pith area, vascular bundle counts, and individual vascular bundle size from digital images of hand-cut transections of stalks collected with a flatbed document scanner. The tool determined vascular bundle number with an average accuracy of 90% across maize genotypes that varied five-fold for this trait. The method is demonstrated on maize, sorghum, and *Miscanthus* stalks. The computer source code is staged for download.

**Conclusions:**

Simplicity of sample preparation and semi-automated analyses enabled by this tool greatly increase measurement throughput relative to standard microscopy-based techniques while maintaining high accuracy. The tool is expected to be useful in genetic and physiological studies of the relationships between stalk anatomy and traits such as biofuel suitability, water use efficiency, or nutrient transport.

## Introduction

The stalks of widely cultivated grass species such as maize and sorghum support multiple architectural and physiological functions, while contributing the most to aerial non-grain biomass. Visible in transections of such stalks are the many vascular bundles scattered throughout the parenchymatous pith. Surrounding the pith is a layer rich in collenchyma that is usually visibly distinct and commonly called the rind. The developmental mechanisms that determine the number of vascular bundles, their distribution, and the proportion of rind to total stem tissue in graminaceous crops is currently an important topic of research [1]. For example, water movement through the plant may relate to the number and size of xylem-bearing vascular bundles. Therefore, selection-based breeding for stem anatomy traits could be an effective strategy to improve water use efficiency [2,3]. Also, cell walls in the sclerenchyma surrounding the bundles or collenchyma in the rind are typically highly lignified, which limits digestibility when the stems are used for animal feedstock or industrial fermentation for ethanol production [4,5]. Plants better suited for ethanol production may be identified through surveys of natural variation for stem anatomy traits [6-8]. Another reason for studying anatomical features of grass stalks is their relationship to mechanical properties. The strength of the stalk determines the degree of wasteful crop lodging and pre-harvest breakage in the field [9-12].

These various motivations for measuring the anatomical features of grass stems create the need for a method that is efficient enough to measure hundreds if not thousands of individuals within defined populations for the purpose of mapping the genetic loci responsible for variation in the trait. Traditional methods for studying anatomy usually rely on sectioning chemically fixed tissue with a microtome followed by mounting the cut section on glass slides for examination with a microscope. These microscopic methods give superb cellular-level resolution, and have been used in large-scale studies of anatomical features [13-15], but typically their throughput is low. Relaxing the resolution criterion from cellular to tissue level increases the feasibility of automation. Higher throughput achieved by greater automation would improve the feasibility of acquiring the large data sets needed for some types of studies, such as statistical genetic trait mapping.

Computerized processing of digital images is an increasingly common means of quantifying plant structure. Sometimes sophisticated microscopy is called for [16-18] but simpler devices such as flatbed document scanners are perfectly adequate in many cases [19,20]. To quantify stem anatomical features as phenotypes across populations of plants suitable for statistical genetic analyses, for example, a simple imaging device and minimal sample preparation may provide the appropriate balance between resolution and throughput. The goal of the present work was to create an image analysis tool that could operate on images of hand-cut stem transections obtained on a flatbed scanner to measure anatomical features in high throughput.

## Results

### Image preprocessing

**Figure 1:**
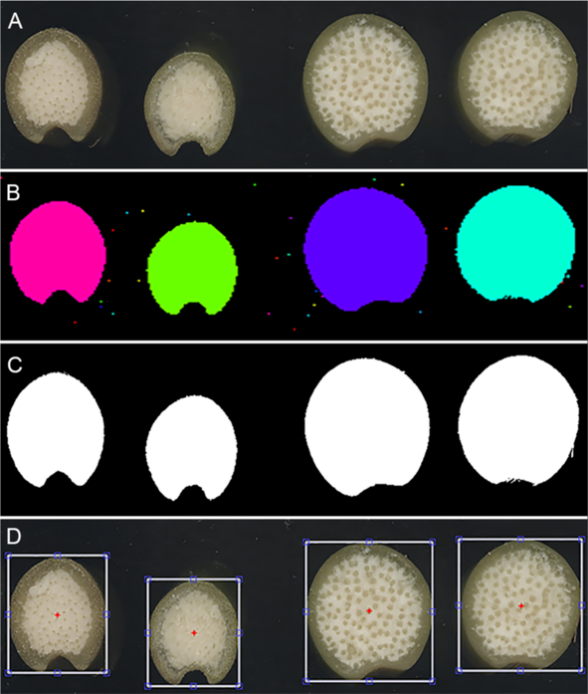
**The initial processing steps of maize stalk transections. A)** Original, unprocessed images of four stalk transections. The two on the left are the same genotype. The two on the right are the same genotype but different from those on the left. **B)** Segmenting all non-background objects by a simple thresholding technique identifies the stalk samples and smaller non-stalk objects such as scratches and debris. **C)** A filtering step based on object size and shape results in a binary image consisting of background and stalk samples. **D)** A crop box centered on each of the stalk objects is placed on the original image to allow the user to make adjustments to the scenes to be processed.

Example images of four stalk transections cut from two different maize genotypes are shown in Figure 1A. The images were acquired with a flatbed document scanner in three-color (red, green, blue) mode. The tool to be described here operates on such images to measure stalk diameter, rind thickness, number of vascular bundles, density of vascular bundles, and vascular bundle size from such images. The first step in the method creates a binary representation of a grayscale version of original image (Figure 1B) using Otsu's method for determining a threshold value that minimizes the intraclass variance [21,22]. A subsequent filtering step eliminates objects (dirt or debris) too small or eccentric (smaller than 15000 pixels, which is significantly smaller than the smallest stalks we have ever analyzed, or with an eccentricity greater than 0.99) to represent a stem section, producing a binary image consisting only of background and stalk transection (Figure 1C). A box bounding each object in the filtered image is automatically placed on the original image for the purpose of cropping individual stem section images (Figure 1D). This box, generated automatically by the program, is the smallest possible rectangle that includes all of the identified object. The user may manually adjust the crop box if, for example, a portion of the stem section should be omitted from further analysis due to damage during sectioning or other anomaly. Standard binary object morphology operations quantify the total area, diameter (average), and perimeter of each stalk section.

The maize genotypes shown in Figure 1 were two of 30 randomly selected from the hundreds of genotypes comprising the Wisconsin Diverse Association Panel [23]. Of the 30 genotypes sampled, E2558W was found to have the widest average stalk diameter and LH85 had the narrowest (Table 1). The difference was approximately two-fold.

**Table 1:** **Range of trait values observed in a sample of thirty maize genotypes**

**Trait**	**Genotype**	**Mean value**
Stalk diameter (cm)	E2558W	3.26 ± 0.23
	LH85	1.60 ± 0.06
Rind thickness (cm)	W182BNgt	0.42 ± 0.08
	A663	0.13 ± 0.04
Vascular bundle density (cm^−2^)	OS602	57.3 ± 3.8
	W182BN	36.8 ± 2.8
Vascular bundle size (cm^2^)	PHK93	9.04 ± 1.1 × 10^−4^
	Mo39	4.31 ± 1.2 × 10^−4^

Of the 30 genotypes sampled by collecting transections from 3 individual plants each, those which gave the largest and smallest value for each trait are listed in the middle column. The mean ± SEM of the three samples per genotype is given in the right hand column.

### Rind thickness

**Figure 2:**
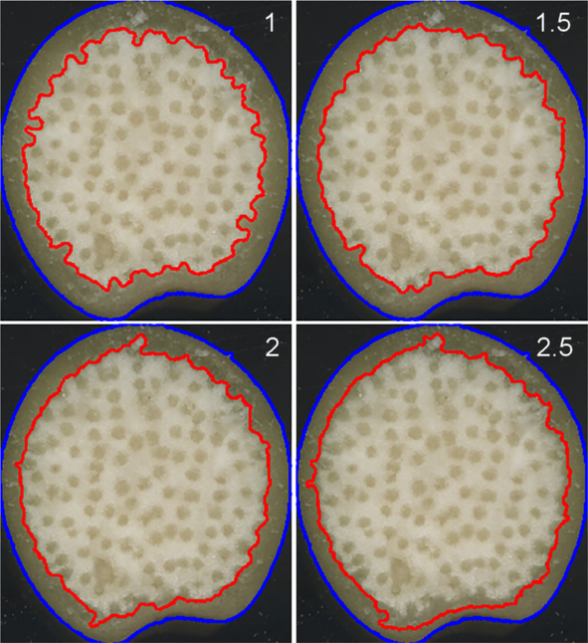
**Discerning the pith/rind boundary.** Processing of the green channel of the original image with a Gaussian filter suppresses the rind region to establish the boundary of the pith (red line). The result depends on the width of the Gaussian filter used to convolve the image. Results from filter width 1, 1.5, 2, and 2.5 are shown.

**Figure 3:**
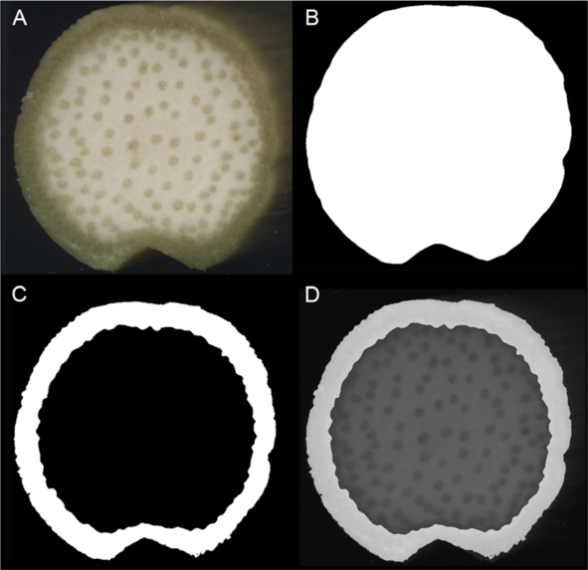
**Determining rind width. A)** An original stalk transection image. **B)** Image after binarization. **C)** Black pith mask determined from boundary found as shown in Figure 2 imposed in the binarized transection to segment the rind (white). **D)** Rind segment overlayed on the image in A. The width of the rind is the average distance between each point on the pith boundary and its nearest point on the sample perimeter.

The rind of monocotyledonous stalks is primarily composed of the epidermis plus lignified sclerenchyma and collenchyma cells beneath the epidermis. To isolate this portion of the stalk from the adjacent central pith, the green channel of the original image was filtered with a Gaussian distribution set to suppress the signal from the rind and maintain the bright signal from the pith. Binarizing this filtered image by Otsu thresholding creates an image with a boundary marked by the red line in Figure 2. The rind segmentation result depends to a relatively minor degree on the filter width parameter, which the user may adjust from the default setting (Figure 2). Figure 3 shows an original transection image, the binarized image, a pith mask based on the step shown in Figure 2, and an overlay showing how the rind could be faithfully segmented from the stalk image. To measure the average width of the rind, the mean Euclidean distance between each point on the object boundary and the point nearest it on the rind/pith boundary is computed. The rind of W182BNgt plants was more than three-fold thicker than that of the A663 genotype (Table 1), indicating considerable genetic variation in the specification of this anatomical trait.

### Vascular bundle number and density

**Figure 4:**
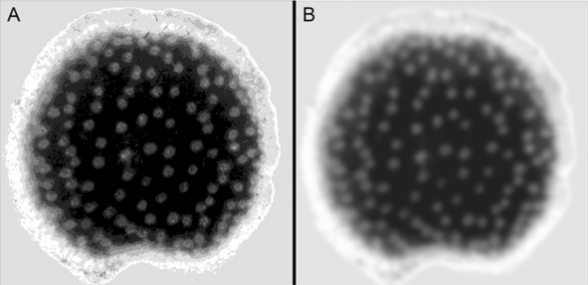
**Processing to highlight vascular bundles.** A grayscale representation of a stalk transection before **(A)** and after **(B)** anisotropic filtering. This step renders the vascular bundles as bright spots of mostly uniform intensity that will appear as single rather than split peaks in a gray value intensity plot.

**Figure 5:**
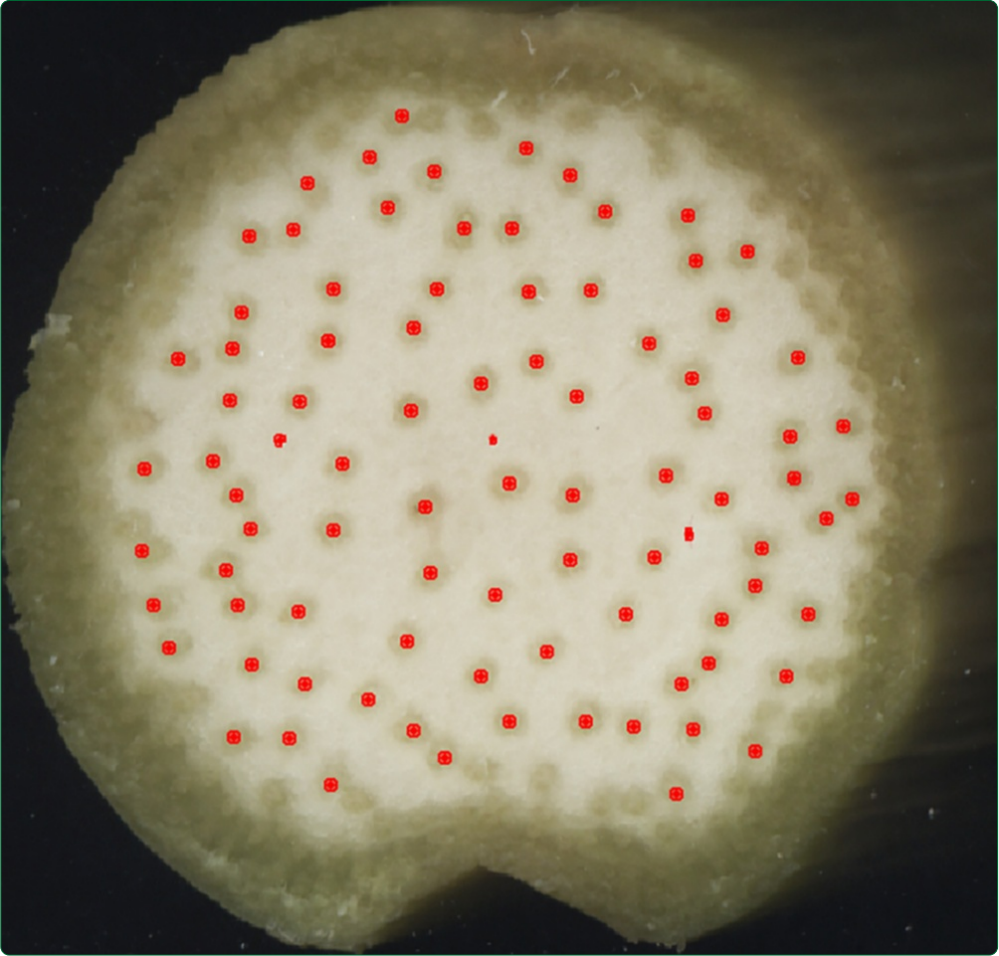
**Vascular bundle localization.** The position of each peak in grayscale value detected after the filtering step shown in Figure 4 is projected on to the original image and labeled in red. At this stage, the user may choose to select bundles not detected by the program.

**Figure 6:**
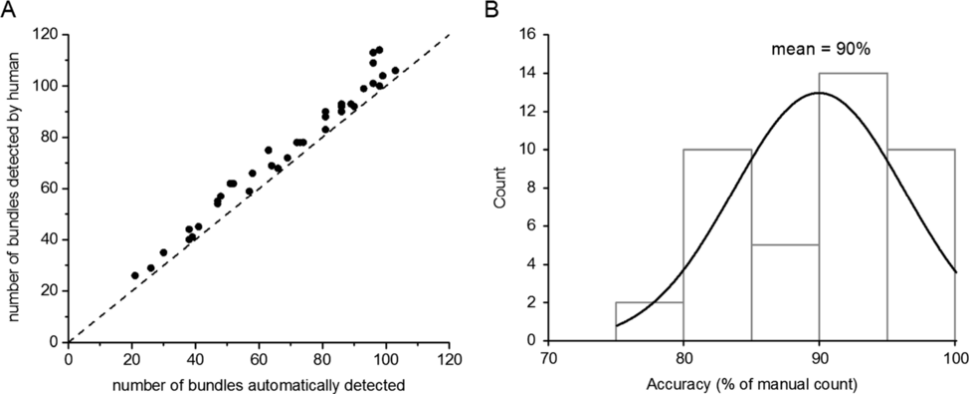
**Accuracy of vascular bundle counting.** A random set of 41 transection images was used to test the accuracy of the tool. **A)** The vascular bundles located in the pith in 41 transections were counted by a human and by the tool. Each point in the scatter plot represents the two numbers associated with each different section. The diagonal line represents perfect agreement. **B)** The number of bundles automatically counted relative to the number determined by eye was converted to a percent accuracy value for each transection and presented as a frequency histogram. Fitting a normal distribution to the histogram determined the mean accuracy value to be 90%.

To enumerate the vascular bundles specifically in the pith, a grayscale version of each image is convolved with a Gaussian kernel having a width approximately equal to the average size of a vascular bundle. A typical result is shown in Figure 4A. An anisotropic diffusion filter [22-26] is then applied to enhance the contrast and reduce noise. These steps result in localized peaks in grayscale value, fairly uniform bright areas, corresponding to the location of vascular bundles (Figure 4B). The peaks are computationally identified via non-maximum suppression [27,28]. Masking the rind (Figure 3) restricts the bundle counts to the pith area. Figure 5 shows a typical result, in which most of the vascular bundles identifiable by eye are counted by the tool. The user may manually select vascular bundles missed by the tool to add to the count. A random sample of 41 transection images was counted by a human expert and processed automatically by the tool. The expert counted 3469 bundles in the piths of these samples and the tool counted 3128, or 90% of the ground truth. Result of this accuracy test are presented in Figure 6. One reason for the consistent though minor undercounting of bundles by the tool is that the human may have included bundles that the tool determined not to be within the pith.

Dividing the number of bundles by the pith area gives vascular bundle density. This trait also varied among the 30 genotypes sampled, though relatively less than the stalk or rind thickness traits. The highest average density, observed in the OS602 genotype, was 57% greater than the lowest density observed, in the W182BN genotype (Table 1).

### Determining the size of the bundles

**Figure 7:**
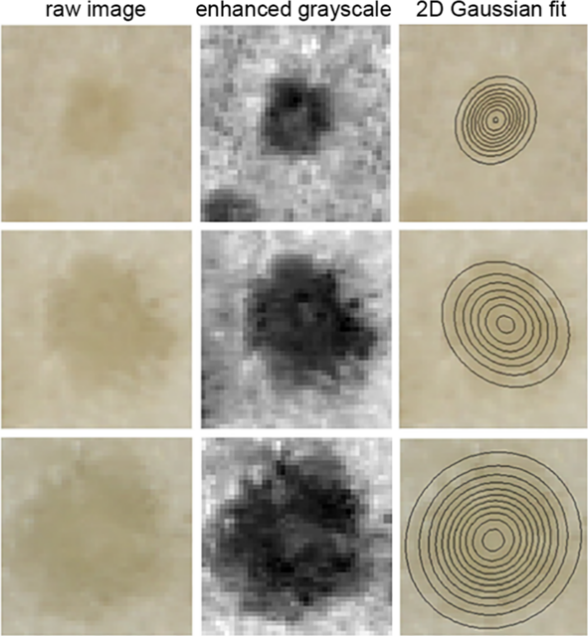
**Measuring vascular bundle size.** Each row is a different individual vascular bundle. A small, medium, and large bundle was selected for presentation. The left column shows the original unprocessed color image. The center column shows the processed grayscale image enhanced by a homomorphic filtering step. The right column shows the level contours of a 2D Gaussian distribution fit to enhanced grayscale map. The outermost ring is taken as a measure of the size of the bundle.

Despite the simplicity of the tissue preparation (hand cut transections) and imaging system (flatbed document scanner) the size of individual vascular bundles could be measured from the images. The process begins by copying a 40 × 40 pixel region surrounding the center coordinates of each previously identified bundle. A homomorphic filtering process [29-31] normalizes brightness and increases contrast of the vascular bundle by first taking the logarithm of the intensity values and then performing two-dimensional discrete Fourier transforms to identify low frequency components of the image, which are subsequently suppressed by filtering because they tend to represent reflectance artifacts more than structural features in images. A 2D Gaussian distribution fit to the grayscale values of this enhanced patch, when projected onto the image plane, produces a measure of the vascular bundle area. Figure 7 shows i) unprocessed vascular bundles of different sizes, ii) after processing, and iii) with contour lines of the fitted Gaussian distribution projected onto the image. Among the genotypes sampled, vascular bundle size varied more than two-fold between the largest in PHK93 and the smallest in Mo39 (Table 1).

### Application to other species

**Figure 8:**
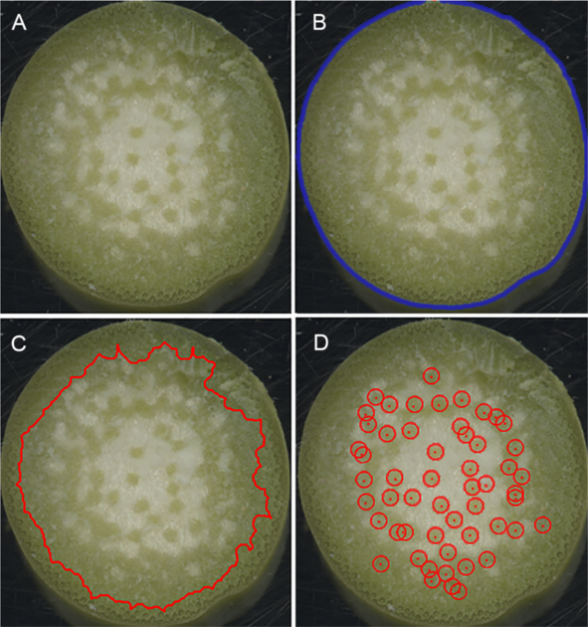
**Applying the tool to transections of** ***Sorghum bicolor*** **stalks. A)** Unprocessed image of a hand-cut transection of a sorghum stalk. **B)** Stalk perimeter (blue line) determined from a binary representation of the transection superimposed on the original image. **C)** Rind/pith boundary (red line) superimposed on the original image. **D)** Detected vascular bundles (red circles) superimposed on the original image.

**Figure 9:**
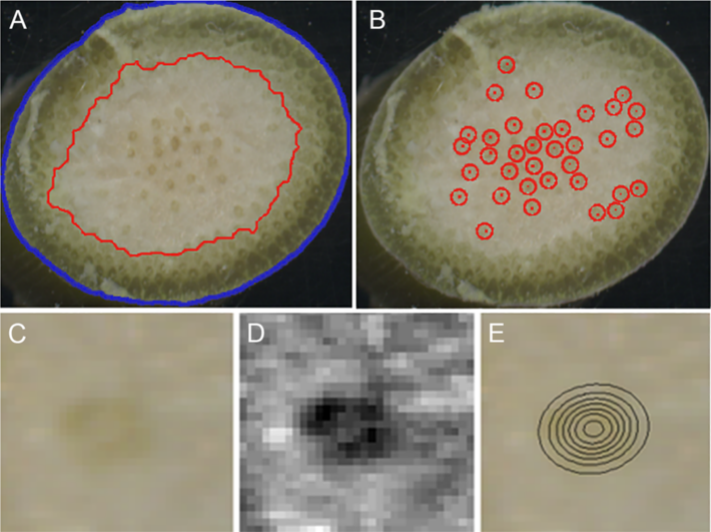
**Applying the tool to transections of** ***Miscanthus gigantum*** **stalks. A)** Stalk perimeter (blue line) and rind/pith boundary (red line) superimposed on the orginial, unprocessed color image. **B)** Detected vascular bundles (red circles). **C)** An individual vascular bundle in the unprocessed image. **D)** Grayscale representation of A after processing including homomorphic filtering. **E)** Level contours of the 2D Gaussian distribution fit to the grayscale image in D projected onto the original image. This shows the method measures the very small vascular bundles in thin stalks of *Miscanthus*.

Transections of *Sorghum bicolor*, a grass species with a maize-like stem anatomy, were similarly collected and imaged as shown in Figure 8A. The tool isolated the boundaries of the rind and enumerated the vascular bundles within the pith (Figure 8B-D). *Miscanthus giganteus*, the stalk of which is intensively studied for biofuels purposes, has considerably narrower stalks and smaller vascular bundles than sorghum or maize. Nonetheless, manually adjusting the respective parameters as discussed above enabled the tool to measure the target suite of traits automatically as demonstrated in maize (Figure 9). Thus, the tool presented here may be expected to quantify anatomical features in the stalks of many grass species having discernable rinds, pith, and vascular bundles.

## Discussion

**Figure 10:**
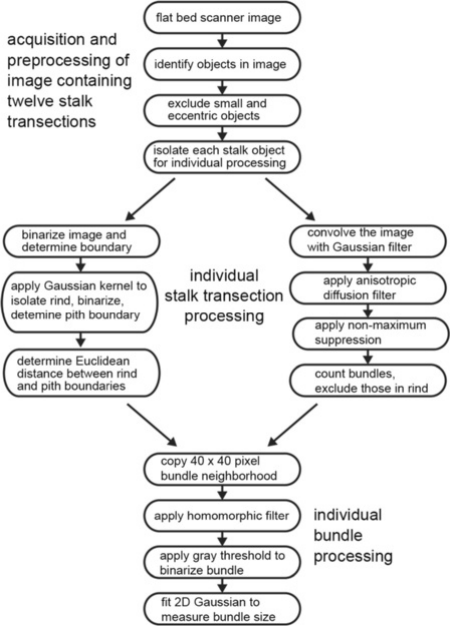
**Flow chart showing the image processing steps from image acquisition to measurement of vascular bundle size.**

The need for a practical method for quantifying the major anatomical features in large numbers of grass crop stalks motivated this tool development project. Three competing objectives needed to be balanced to meet the need. One objective was to require only minimal sample preparation. Another was for the analysis not to limit the rate of the overall process. The third was to achieve high accuracy. Sample preparation involves hand cutting transections from stalk internodes systematically collected and tagged in the field and placing them directly on the imaging surface of a computer-controlled document scanner. Image acquisition and file saving requires only one manual input. Thus, the actions needed to prepare and image the samples are simple and efficient. Analysis of the resulting image file representing 12 transections is typically automatic, though scenarios requiring manual adjustments are encountered. For example, regions physically damaged during sectioning or by insect feeding may require cropping by manually adjusting the bounding box. The default settings of the Gaussian distribution width or the rigidity of the anisotropic diffusion parameters serve well for most maize transections but may require adjustment when strongly divergent genotypes or other species are to be studied. Even in scenarios requiring semi-automated operation, in which the user chooses some degree of supervision and parameter adjustment, the analysis step was not more time consuming than sample preparation. Achieving acceptable accuracy or fidelity in the analysis stage required development of appropriate feature extraction algorithms. Here the technical challenges to be overcome stemmed principally from the choice of simple sample preparation and imaging because without fixation, staining, or optical enhancement of contrast, the anatomical information in the images is frequently obscure. The steps described here and summarized in a flowchart (Figure 10) dealt with the inhomogenieties in lighting across individual transections, probably due to variation in sample thickness and other consequences of crude hand sectioning techniques, better than alternative image processing methodologies such as watershed [32,33] and level-set techniques [34,35], which were also tried. Lack of sample uniformity probably had a larger effect on result quality than did parameter tuning. Therefore, better methods for stalk sectioning may have a larger impact on the capability of this tool than further software efforts.

Overall, the set of design decisions and technical solutions produced an effective tool for high-throughput quantification of anatomical features in grass stalks. The tool, written in the Matlab computer language, is staged for download at http://phytomorph.wisc.edu/download/HeckwolfPlantMethods2015/ along with a composite test image representing a variety of transection phenotypes so that the performance of future tools for studying stalk anatomy can be benchmarked against that described here.

The current tool and future derivatives of it may be expected to enable systems-style projects attempting to discover links between the anatomical features and chemical or gene expression features, or large-studies of the genetic architecture affecting stalk architecture. More specifically, this tool may benefit biofuels researchers seeking a deeper understanding of the relationships between stalk anatomy, cell wall composition, and efficiency of carbohydrate conversion to ethanol because lignin content, which is correlated with features such as vascular bundle density quantified here (Table 1), limits the efficiency of biomass to ethanol conversion. Modeling of the size and number of vascular bundles in grass stalks for the purpose of achieving a quantitative understanding of stalk hydraulics is another area of research that may benefit from the tool.

This motivation for the development reported here, and therefore the resulting product, differs from a recent study that also used image analysis of maize stalk transections but to produce a 3D statistical model of vascular bundle distribution [36]. The present work emphasizes measurement throughput, to address the need for quantifying the most salient anatomical features in thousands of stalk samples, whereas Legland et al. [36] created a normalized model of vascular bundle distributions that facilitates quantitative comparisons between categories of samples.

Although the tool was developed to be effective with minimal sample preparation, it may prove useful in studies of transections that have been stained to highlight features such as high lignin content. Incorporation of such labeling or staining steps may give the method reported here more power to resolve anatomical details, for example by differentiating between sclerenchyma and collenchyma within the rind [37,38].

## Methods

### Plant growth and sample collection

A set of 30 diverse maize inbred lines from the Wisconsin Diverse Association Panel [23], was grown at the Arlington Agricultural Research Station (University of Wisconsin-Madison) in 2013 in a field experiment using a randomized complete block design with two replications. Three representative plants per plot and field replication were harvested and brought to the lab 45 days after flowering for sample preparation.

### Sample preparation and imaging

Transections of the third internode above the ground were cut by hand using razor blades into sections between 4 and 10 mm thick. Sections thicker than that resulted frequently in shadows or reflections on the rim of the stalk, producing a halo effect, which made the analysis of the stalks using standard parameter setting difficult or impossible. To produce an image for analysis, a total of 12 transections representing three individuals of four genotypes were placed on the horizontal imaging surface of an Epson Perfection V700 Photo Scanner and scanned at resolution of 800 dots per inch in red, green, blue color mode. Using these settings, we produced images with a height of 3800 to 3900 pixels and a width of 5000 to 5100 pixels and a resolution of 315 pixels per centimeter, with an error of at most 5 pixels. To enhance contrast between the samples and the background, the lid of the scanner was left open, resulting in a black background. The resulting files, typically larger than 30 megabytes, were saved in tagged image file format to a computer disk array.
